# Ethnic Discrimination’s Role on Increased Substance Susceptibility and Use Among U.S. Youth

**DOI:** 10.1016/j.amepre.2025.107956

**Published:** 2025-06-25

**Authors:** Robert Rosales, Philip T. Veliz, John Jardine, Alexander S. Weigard, Sean Esteban McCabe

**Affiliations:** 1 Center for Alcohol and Addiction Studies, Department of Behavioral & Social Science, School of Public Health, Brown University, Providence, Rhode Island;; 2 Department of Systems, Populations and Leadership, University of Michigan School of Nursing, Ann Arbor, Michigan;; 3 Applied Biostatistics Lab, University of Michigan School of Nursing, Ann Arbor, Michigan;; 4 Addiction Center, Department of Psychiatry, Michigan Medicine, University of Michigan, Ann Arbor, Michigan;; 5 Center for the Study of Drugs, Alcohol, Smoking and Health, Department of Health Behavior and Biological Sciences, University of Michigan School of Nursing, Ann Arbor, Michigan

## Abstract

**Introduction:**

Recently, U.S. youth of color reported greater use of alcohol, tobacco, and cannabis than White youth. Increased levels of discrimination in recent years may have added to the chronic burden associated with increased use among youth of color. Little is known about this relationship, especially among youth who initiate substance use earlier in adolescence. This study assessed the prevalence of substance susceptibility (willingness and curiosity) and use (alcohol, tobacco, and cannabis) among youth by race/ethnicity and ethnic discrimination’s role in this relationship.

**Methods:**

Data from the national panel of 11,868 U.S. youth in the Adolescent Brain Cognitive Development study (baseline through fourth year follow-up; 2016–2022), which assessed these relationships beginning at ages 9–10 years, were analyzed in 2024–2025. Prevalence of lifetime substance susceptibility and use were quantified by race/ethnicity. Multivariable longitudinal analyses tested whether discrimination was connected to substance susceptibility and lifetime use and whether that relationship differed by race/ethnicity.

**Results:**

Black youth reported lower lifetime alcohol and tobacco use, lower curiosity toward alcohol and tobacco, and higher willingness to try alcohol than White youth. Hispanic youth reported higher willingness to try alcohol. Asian youth reported lower lifetime tobacco use. Higher levels of ethnic discrimination were consistently associated with greater odds of susceptibility and use among all racial/ethnic groups in this study.

**Conclusions:**

Results show that youth of color report lower substance use; however, ethnic discrimination may account for some of the recent increased national trends in substance use among youth of color through its impact on their increased susceptibility to use substances.

## INTRODUCTION

Substance use disorders (SUDs) cause a significant health burden worldwide. In the U.S. alone, more than 65 million adults will have an alcohol use disorder in their lifetime, 8.5% of adults report a nicotine dependence in the past month, 6.8% reported a cannabis use disorder in the past year, and 9.6% of adults report any drug use disorder in the past year.^1,2^ Tobacco use remains the leading cause of preventable disease, disability, and death, with approximately 500,000 tobacco-related deaths each year.^3,4^ In past years, the major burden of SUDs has been concentrated mostly among White people in the U.S.^5,6^ However, Black and Hispanic youth in the 2022–2023 Monitoring the Future studies reported that they are more likely to use most substances (e.g., alcohol, cannabis, and tobacco) than White youth, a reversal from years prior to 2022.^5,6^ Similarly, higher prevalence of tobacco use among youth of color than among White youth has been found in the 2023–2024 National Youth Tobacco Survey.^7^ This shift in substance use prevalence may signal future racial differences in SUDs as youth age into adulthood. Identifying the predictors of this increased risk for substance use among youth of color, especially youth who initiate earlier in adolescence and are therefore at increased risk for future negative outcomes,^8,9^ may help determine the mechanisms that need to be addressed in prevention.

Stress theories posit that discrimination creates a chronic stress burden that accumulates over time to diminish a person’s ability to cope, making them more susceptible to substance use.^10^ Prior research supports this theory, showing that greater discrimination is associated with greater substance use among youth of color.^11–17^ One longitudinal study with Black adolescents in the U.S. found that discrimination among individuals aged 10–12 years was associated with problematic substance use nearly 5 years later.^18^ Among Hispanic adolescents in Los Angeles, discrimination in 9th grade has been shown to predict greater substance use in 11th grade.^19^ In addition, a longitudinal study with 350 teenagers in the northeast showed that greater discrimination was related to greater past-year alcohol use among Asian teenagers; meanwhile, multiracial teenagers experiencing greater discrimination reported greater past-month alcohol use 12 months later.^20^ However, it is unknown whether the recent trends in substance use are also associated with discrimination. It is possible that people of color’s heightened stress in recent years (e.g., during the coronavirus disease 2019 [COVID-19] pandemic) may partially explain the increase in substance use.^21,22^ Youth of color may have used substances at a greater prevalence in recent years because of the recent increases in discrimination they experienced in early childhood.

Most recent Monitoring the Future data show that differences in substance use between youth of color and White youth are apparent in 10th grade.^5,6^ Attitudes toward using substances may help to identify youth who have a higher susceptibility to using substances in the future.^23,24^ It has been hypothesized that susceptibility to use substances can translate into established substance use within 2 years.^23^ Susceptibility to substance use, such as curiosity and willingness to try substances, can predict future substance use behavior beyond other risk factors.^23–30^ Youth of color report greater susceptibility to using substances,^29^ which suggests that identifying the underlying mechanisms driving greater susceptibility may help to prevent the continued high risk of substance use among youth of color seen in recent trends. In addition, willingness and curiosity to try substances have been shown to predict future use, including among Hispanic youth.^31–35^ Substance use risk can be assessed prior to 10th grade by measuring these key precursors.

Little is known about the relationship between ethnic discrimination and susceptibility to use,^36^ especially among youth of color in late childhood and early adolescence, despite the importance of these developmental stages to understanding precursors to substance use. However, some studies have shown preliminary findings.^18,37–40^ A longitudinal study with Black youth showed that discrimination in early childhood was associated with intentions to use substances 2 years later.^18^ Black youth who experienced multiple types of discrimination also report more intentions to use substances in the future, but this was not the case for Hispanic or multiracial youth.^38^ Assessing the role of ethnic discrimination in the susceptibility of substance use during childhood may provide insights on the growing substance use prevalence among youth of color.

This study used data from one of the largest national publicly available data sets of adolescent health (Adolescent Brain Cognitive Development [ABCD]) to assess (1) the prevalence of substance susceptibility and use, (2) whether ethnic discrimination is related to substance susceptibility and use, and (3) whether these relationships differed by race/ethnicity.

The goal was to determine whether the recent turning point in substance use among youth of color is associated with ethnic discrimination and whether it plays a role in early substance susceptibility and use among a sample as young as individuals aged 9 years.

## METHODS

### Study Sample

Secondary data analyses of the ABCD data were conducted. The ongoing ABCD study has collected repeated-measures responses from 11,868 youth recruited nationally over 10 years using probability sampling starting at age 9 years.^41^ The ABCD study used a multistage probability sampling of public and private schools within 21 catchment sites to approximate the demographic and geographic diversity of U.S. adolescents. This study used the publicly available panel data at the start of this study’s data analysis (September 2024), including baseline to 4th follow-up (2016–2022). Because the ABCD is not intended to be nationally representative, it does not include population or attrition weights. Owing to the ABCD data being publicly available and deidentified, this study was exempt from Brown University’s IRB review. Appendix Table 1 Study Sample (available online) provides more information.

### Measures

Perceived Ethnic Discrimination (PED) Scale is a 7-item measure created to assess perceptions of ethnic discrimination among minority and immigrant youth using items such as *Others behave in an unfair or negative way toward my ethnic group* and *I don’t feel accepted by other Americans*.^42^ The PED, given to all youth in the study, uses a 5-point scale ranging from 0 (almost never) to 4 (very often) and has shown high reliability in past research (e.g., α=81–90).^42,43^ Youth participant’s responses were averaged, with higher responses indicating greater PED. Because this item was not measured at baseline and the year 3 follow-up, respondents’ highest scores across the Years 1, 2, and 4 follow-ups were used to avoid missingness. Responses by wave can be found in Appendix Table 2.

Substance use was evaluated at baseline (lifetime use) and each follow-up event (use since the last session was completed) using the substance use interview and substance use phone interview (mid-year) modules. For alcohol, ever use is defined as having tried a full drink of beer, wine, or liquor. For tobacco and marijuana, ever use is defined as having tried any tobacco/nicotine or marijuana product, including just a puff. More details are given in the table footnotes.

Two items were used from the Susceptibility to Use Index to measure susceptibility to use tobacco, alcohol, and marijuana.^23,24^ The curiosity items were measured with 3 questions prompted with *Have you ever been curious about…: using a tobacco product such as cigarettes, e-cigarettes, hookah, or cigars?, drinking alcohol?*, and *trying marijuana?* These measures were dichotomized such that 0=not at all curious and 1=a little curious to very curious. The willingness to try items was also measured with 3 questions prompted with *Do you think you will try…: a tobacco product soon?, alcohol soon?, and marijuana soon?* These measures were dichotomized such that 0=definitely not/probably not and 1=definitely yes/probably yes. The Susceptibility to Use Index has been shown to be a valid and reliable measure of substance use among youth of color. Responses by wave can be found in Appendix Table 3.^44^

Race/ethnicity was measured with 2 items that asked the parent, *What race do you consider the child to be?* and *Do you consider the child Hispanic/Latino/Latina?* The original race variable, derived from a PhenX item, included the following responses: (1) *White*; (2) *Black/African American*; (3) *American Indian, Native American*; (4) *Alaska Native*; (5) *Native Hawaiian*; (6) *Guamanian*; (7) *Samoan*; (8) *Other Pacific Islander*; (9) *Asian Indian*; (10) *Chinese*; (11) *Filipino*; (12) *Japanese*; (13) *Korean*; (14) *Vietnamese*; (15) *Other Asian*; (16) *Other Race*; (17) *Refuse*; and (18) *Don’t Know*.^45^ Respondents were also asked to report whether they considered that their children were Hispanic/Latino/Latina with the following responses: *Yes, No*.^45^ ABCD recoded these responses into a single, 5-category variable: (1) White, (2) Black, (3) Hispanic, (4) Asian, and (5) other race or multiracial.

### Statistical Analysis

All data analyses were conducted in Stata 18.0. Bivariate analyses were conducted to assess the prevalence of substance susceptibility and use by race/ethnicity. Generalized estimating equations (with an exchangeable correlation matrix) were used to assess the longitudinal association between race/ethnicity, ethnic discrimination and (1) substance use, and (2) susceptibility to use, where substance use and susceptibility to use were measured as time varying. Models assessing discrimination scores for each race (i.e., discrimination scores for each specific race) were then conducted to assess how the level of discrimination for each race/ethnicity (i.e., non-Hispanic White only, non-Hispanic Black only, Hispanic, non-Hispanic Asian only, non-Hispanic other race, or multiracial) was differentially associated with substance susceptibility and use. Each model was conducted separately to assess the outcome, which included 9 models for main effects (Models 1–9) and 9 for discrimination scores by race/ethnicity (Models 10–18). Note that standard interaction effect models were not used owing to sample size issues (several models were not able to estimate)—these interaction effect models are provided in Appendix Tables 4 and 5 (available online) and confirm the findings from Models 10–18. Covariates were measured at baseline, except discrimination, which represents the maximum level of discrimination endorsed by the participant across the Years 1, 2, and 4 follow-ups. Models were adjusted for time, the respondent’s age at baseline, the respondent’s sex assigned at birth (from hereon referred to sex), parental marital status, parental education level, parental employment status, and total combined family income (all variables were treated as categorical). Statistical significance was set at 0.05 alpha level. All models used listwise deletion.

## RESULTS

Table 1 provides the sample characteristics for the adolescents in the sample. At baseline, respondents were mostly aged 9 years (52.6%) and male (52.2%). The sample included 1,784 non-Hispanic Black (15%; Black); 2,410 Hispanic (20.3%); 252 non-Hispanic Asian (2.1%; Asian); 1,247 non-Hispanic other/multiracial (10.5%; other/multiracial); and 6,173 non-Hispanic White (52.1%; White) participants. In terms of family makeup, most participants’ caregivers had a bachelor’s degree or higher (59.5%); were married (67.8%); worked full time (84.8%); and made ≥$100,000 total combined family income (38.4%).

Table 2 presents the prevalence of ever using substance and susceptibility by race/ethnicity. White youth reported the highest prevalence of ever using alcohol and curiosity about alcohol. Black youth reported the highest prevalence of being willing to try alcohol, tobacco, and marijuana. Hispanic youth reported the highest prevalence of ever using marijuana. Other/multiracial youth reported the highest prevalence of ever using tobacco, curiosity about tobacco, and curiosity about marijuana.

Table 3 presents the main effects of race/ethnicity and ethnic discrimination on lifetime substance use and susceptibility. Higher levels of ethnic discrimination were consistently associated with greater odds of use, curiosity, and willingness to use substances among all substances in this study (AOR range=1.61–2.08). Black youth reported lower lifetime use of alcohol (AOR=0.26) and tobacco (AOR=0.44), lower curiosity toward alcohol (AOR=0.76) and tobacco (AOR=0.74), and higher willingness to try alcohol (AOR=1.83) than White youth; Hispanic youth only reported higher willingness to try alcohol (AOR=2.03); and Asian youth only reported lower lifetime tobacco use (AOR=0.11).

The models assessing the level of discrimination for each race/ethnicity and substance use showed that only White, Hispanic, and other/multiracial youth reported greater substance use odds when facing higher levels of discrimination (AOR range=1.60–2.48). The odds of curiosity for level of discrimination for each race/ethnicity were uniformly positive across all groups, except for Asian youth (AOR range=1.21–2.02). Black and other/multiracial youth reported higher odds of willingness to use substances when experiencing discrimination (AOR range=1.66–2.58). Discrimination was related with greater willingness to try tobacco for White (AOR=1.87), alcohol and cannabis for Hispanic (AOR =1.77), and alcohol for Asian (AOR range=1.66–2.58) youth. More detailed findings are presented in Appendix Table 6 (available online).

## DISCUSSION

Owing to the high prevalence of substance use among racial/ethnic minorities in recent years, this study assessed substance use prevalence and associations between ethnic discrimination experiences and substance susceptibility and use among racial/ethnic youth groups. To the authors’ knowledge, this study provides a unique contribution to the literature by being one of the first studies to assess these associations in youth of color who are in their late childhood and early adolescence. Given the well-documented association of early substance use initiation with negative outcomes,^8,9^ the findings provide novel insights into these processes in a developmental period that is critical for understanding precursors to problematic substance use. The findings reinforced some of the results from other national studies showing higher substance use among Hispanic and Black youth than among White youth.^5,6^ This study found that Black, Hispanic, and other/multiracial youth reported greater tobacco and cannabis use than White youth. Other/multiracial youth had the highest prevalence of ever using any substance, consistent with a growing body of research.^46–48^ However, prevalence of cannabis use was small, and the disparities in substance use were not statistically significantly greater after adjusting for other variables. In multivariate models, White youth had significantly higher prevalence of ever using alcohol and tobacco than Black youth.

Although White youth reported the highest prevalence of ever using alcohol, they reported lower willingness to try alcohol soon than Black and Hispanic youth. Most of the ABCD sample in the fourth wave have not reached 10th grade yet, suggesting that the majority of alcohol use initiation will occur at subsequent time points as youth transition from middle school to high school.^5,6^ Thus, Black and Hispanic youth’s increased willingness to try alcohol may point to greater future alcohol initiation in these groups, leading to future increases in disparities related to alcohol use. In addition, youth of color report greater susceptibility to using substances,^29^ further indicating that addressing susceptibility may help prevent the increased onset seen among youth of color.

Black youth reported significantly lower curiosity toward alcohol and tobacco than White youth. Research shows that family and cultural factors protect Black youth from using substances.^49–51^ Spending time with family, talking to parents about problems related to ever using substances, and having parents who disapprove of their child using substances help Black youth abstain from using substances.^50^ Black youth may be more attached to their parents and have parents who have more proactive parenting styles than White youth, which could help with monitoring of youth’s substance use.^51^

Other/multiracial youth reported the greatest prevalence of curiosity toward tobacco and cannabis than all other racial/ethnic groups in this study. It is possible that the high risk seen in research related to substance use among multiracial youth may be related to greater curiosity toward these substances.^46–48^ However, this finding was not significantly different from that among White youth when adjusting for covariates.

The findings related to ethnic discrimination support models suggesting that discrimination is associated with increased odds of ever using substances, curiosity, and willingness to use across all racial/ethnic groups.^10^ With the exception of willingness to use cannabis and alcohol, the findings show that White youth report odds of lifetime substance susceptibility and use when discrimination scores increased. In addition, some of the racial/ethnic groups of youth reported high odds of willingness to use certain substances when experiencing greater discrimination. Asian youth reported higher odds of willingness to use alcohol when faced with discrimination. This finding may be an early signal for heavy episodic drinking seen in Asian adult populations in the U.S.^52,53^ Especially in recent years and during the COVID-19 pandemic, there have been heightened levels of discrimination that have been associated with high heavy episodic drinking among this population.^53,54^ These levels of discrimination and other factors, such as acculturation, may put Asian youth at a heightened susceptibility to using alcohol to cope with discrimination when the cumulative effects of discrimination have exhausted their other coping resources.^53,55–57^ This finding may suggest that discrimination will present as a substantial reason why Asian youth may try alcohol as they age. These findings with Asian youth should be interpreted cautiously owing to the wide CIs.

Black, Hispanic, and multiracial youth also reported greater odds of being willing to try cannabis when faced with greater discrimination. These findings support prior research showing that Black, Hispanic, and multiracial youth report high levels of initiating cannabis use at an early age and using cannabis when experiencing discrimination.^58–60^ Discrimination may decrease various biological, including neurobiological, resources that would otherwise help protect youth of color from using and intending to use substances.^61^

The finding related to discrimination and substance susceptibility and use signals that substance use differences may widen. Research has consistently suggested that there are protective factors, such as ethnic identity salience and familism, that can protect youth of color from the negative effects of discrimination.^62–65^ However, experiences of discrimination can have a cumulative effect, which can erode youth of color’s protective maneuvers over time and produce worse outcomes than among White youth.^10,66,67^ If the findings related to discrimination and susceptibility to use translates to future use, the effects of discrimination on substance use may have a stronger effect for youth of color beyond the 10th grade.

### Limitations

This study is not without limitations. First, youth from heterogeneous racial/ethnic groups were combined into 5 groups. Future studies should assess whether country of origin, skin color, and other differences within racial/ethnic groups may be related to variance in these relationships. Second, substance use is still relatively low in this young cohort, making it difficult to assess differences across racial/ethnic groups owing to sample size constraints, including American Indian or Alaska Native youth. Future studies with later ABCD follow-ups will need to be assessed to track growing racial/ethnic disparities in substance use. Third, baseline measurement of parental demographic variables (e.g., marital status) were used even though these factors could change over time, especially during the COVID-19 pandemic. The posthoc analyses (Appendix Table 3, available online) of these variables showed that they were mostly stable over time at a rate <2% change per follow-up. Fourth, this study focused on 1 important type of discrimination (ethnic), and future work is warranted on other types of discrimination (e.g., sex). Relatedly, because discrimination was averaged over multiple time waves, the temporal sequence between discrimination experiences and substance susceptibility and use is uncertain. Finally, there is potential reporting bias related to youth’s understanding of the measures in this study, especially in the first wave of the study when they are younger. Although the PED measured experiences of discrimination related to ethnicity, some youth may have reported discriminatory experiences related to other identities.

## CONCLUSIONS

Findings both support and extend previous findings by showing that ethnic discrimination is associated with greater substance use risk across U.S. racial/ethnic groups during ages 9–14 years. Ethnic discrimination was associated with substance susceptibility among some youth of color compared with that among White youth, presenting as a potential public health issue for these groups. Thus, future research should assess the mediators related to substance susceptibility and use among separate racial/ethnic subgroups. These findings may motivate efforts to address and prevent recent racial differences among U.S. youth before they transition into later adolescence and adulthood.

## Supplementary Material

1

Supplemental materials associated with this article can be found in the online version at https://doi.org/10.1016/j.amepre.2025.107956.

## Figures and Tables

**Table 1.** Sample Characteristics of Respondents in the ABCD Study (N=11,868)

Variable	Response category	*n* (%)	Missing
Child’s age at baseline, year	9 or younger	6,238 (52.57%)	1 (0.01%)
	10 or older	5,629 (47.43%)	
Child’s sex	Male	6,191 (52.17%)	0 (0%)
	Female	5,677 (47.83%)	
Child’s race/ethnicity	Non-Hispanic White only	6,173 (52.02%)	2 (0.02%)
	Non-Hispanic Black only	1,784 (15.03%)	
	Hispanic	2,410 (20.31%)	
	Non-Hispanic Asian only	252 (2.12%)	
	Non-Hispanic other race or multiracial	1,247 (10.51%)	
Highest level of parental education	Less than high school	433 (3.65%)	14 (0.12%)
	High school	1,292 (10.90%)	
	Some college	1,505 (12.70%)	
	Associate’s degree	1,569 (13.24%)	
	Bachelor’s degree or higher	7,055 (59.52%)	
Parental marital status	Not married	3,789 (32.19%)	96 (0.81%)
	Married	7,983 (67.81%)	
Parental employment status	Neither parent or partner works full-time	1,790 (15.20%)	94 (0.79%)
	At least one of parent or partner works full-time	9,984 (84.80%)	
Total combined family income	≤$24,999	1,634 (13.77%)	2 (0.02%)
	$25,000–$49,999	1,588 (13.38%)	
	$50,000–$74,999	1,498 (12.62%)	
	$75,000–$99,999	1,570 (13.23%)	
	≥$100,000	4,561 (38.44%)	
	Don’t know	504 (4.25%)	
	Refuse to answer	511 (4.31%)	
Discrimination score	x=0	5,436 (47.56%)	439 (3.70%)
	0<x≤0.5	3,672 (32.13%)	
	x>0.5	2,321 (20.31%)	

*Notes:* All measures except the discrimination item were assessed at baseline. The discrimination item represents the respondent’s highest score across the Years 1, 2, and 4 follow-ups (assuming that they had a nonmissing value for at least 1 event). The discrimination item was rescaled from 1–5 to 0–4.

ABCD, Adolescent Brain Cognitive Development.

**Table 2.** Prevalence of Substance Susceptibility and Use by Race/Ethnicity: % (95% CI)

				Susceptibility to use					
	Substance use			Curiosity			Will try soon		
Race/ethnicity	Alcohol	Tobacco	Cannabis	Alcohol	Tobacco	Cannabis	Alcohol	Tobacco	Cannabis
Non-Hispanic White only	3.76 (3.30, 4.28)	5.28 (4.72, 5.91)	2.80 (2.41, 3.25)	36.13 (34.69, 37.60)	30.03 (28.85, 31.24)	15.10 (14.18, 16.07)	2.73 (2.29, 3.25)	1.52 (1.24, 1.87)	1.17 (0.93, 1.48)
Non-Hispanic Black only	1.12 (0.72, 1.73)	4.76 (3.87, 5.85)	3.48 (2.71, 4.45)	26.46 (24.23, 28.81)	25.62 (23.50, 27.87)	15.31 (13.61, 17.19)	6.13 (5.03, 7.44)	3.65 (2.86, 4.64)	3.47 (2.69, 4.47)
Hispanic	2.78 (2.17, 3.55)	6.02 (5.08, 7.11)	3.90 (3.18, 4.79)	35.11 (32.97, 37.31)	30.78 (28.91, 32.72)	14.67 (13.25, 16.21)	5.73 (4.75, 6.89)	2.76 (2.15, 3.52)	2.10 (1.57, 2.79)
Non-Hispanic Asian only	0.40 (0.06, 2.79)	0.79 (0.20, 3.14)	0.79 (0.20, 3.14)	32.47 (26.31, 39.31)	27.76 (22.37, 33.87)	10.68 (7.10, 15.76)	4.62 (2.40, 8.68)	0.40 (0.06, 2.84)	0.43 (0.06, 3.02)
Non-Hispanic other race or multiracial	3.05 (2.21, 4.19)	7.54 (6.16, 9.19)	3.53 (2.62, 4.74)	32.58 (29.59, 35.72)	32.16 (29.54, 34.89)	18.26 (16.13, 20.60)	4.11 (2.99, 5.63)	2.95 (2.14, 4.06)	2.16 (1.47, 3.15)

*Notes:* For calculating prevalence of substance susceptibility and use, respondents were coded as 1 if they indicated use at any event. Else, they were coded as 0 as long as they indicated no use at 1 event at least. For the models in Table 2, substance susceptibility and use were left as time varying. For alcohol, use is defined as having tried a full drink of beer, wine, or liquor. For tobacco, use is defined as having tried a tobacco cigarette; an E-cigarette, vape pen, or electronic hookah; smokeless tobacco, chew, or snus; cigars, including traditional cigars, little cigars, or cigarillos; hookah; pipes; and nicotine replacements, such as patches, gums, nasal sprays, inhalers, and lozenges. A puff of a tobacco product counts as use. For marijuana, use is defined as having tried smoking marijuana; blunts; marijuana that you eat, such as pot cookies, gummy bears, or brownies; marijuana oils or concentrates; marijuana-infused alcohol drinks; concentrated marijuana tinctures; vaping marijuana flower or bud; and vaping marijuana oils or concentrates. More detailed examples of certain methods are given in the ABCD codebooks. A puff of a marijuana product counts as use.

For a given event, only respondents who knew what the substance was but had not tried it yet (including just a sip of alcohol or a puff of tobacco/marijuana) were asked about their curiosity and willingness to try that substance.

ABCD, Adolescent Brain Cognitive Development.

**Table 3.** AORs for Race/Ethnicity and Discrimination on Substance Susceptibility and Use: AOR (95% CI)

	Substance use			Susceptibility to use					
				Curiosity			Will Try Soon		
	Alcohol *n*=11,257	Tobacco *n*=11,257	Cannabis *n*=11,257	Alcohol *n*=8,657	Tobacco *n*=11,122	Cannabis *n*=10,905	Alcohol *n*=8,660	Tobacco *n*=11,121	Cannabis *n*=10,905
Effects	obs.=47,834	obs.=47,834	obs.=47,831	obs.=32,474	obs.=40,713	obs.=32,714	obs.=32,421	obs.=41,005	obs.=32,794
Main effects: Models 1–9									
Non-Hispanic White only	ref	ref	ref	ref	ref	ref	ref	ref	ref
Non-Hispanic Black only	**0.26** (0.11, 0.59)	**0.44** (0.27, 0.71)	0.70 (0.40, 1.22)	**0.76** (0.61, 0.94)	**0.74** (0.60, 0.92)	0.81 (0.61, 1.06)	**1.83** (1.08, 3.10)	1.19 (0.63, 2.22)	1.59 (0.83, 3.06)
Hispanic	0.76 (0.45, 1.28)	0.78 (0.54, 1.14)	0.95 (0.59, 1.53)	1.12 (0.94, 1.33)	0.97 (0.82, 1.14)	0.84 (0.66, 1.07)	2.03 (1.25, 3.29)	1.11 (0.61, 2.04)	1.05 (0.55, 2.01)
Non-Hispanic Asian only	0.08 (0.00, 1.47)	**0.11** (0.01, 0.87)	0.26 (0.03, 2.16)	0.81 (0.53, 1.23)	0.92 (0.60, 1.39)	0.62 (0.32, 1.21)	2.18 (0.72, 6.57)	0.30 (0.02, 5.74)	0.37 (0.02, 7.14)
Non-Hispanic other race or multiracial	0.69 (0.39, 1.24)	1.01 (0.67, 1.53)	0.94 (0.54, 1.63)	0.98 (0.79, 1.21)	1.03 (0.85, 1.25)	1.21 (0.93, 1.56)	1.46 (0.81, 2.61)	1.37 (0.71, 2.63)	1.41 (0.67, 3.00)
Discrimination score	**1.92** (1.46, 2.52)	**1.88** (1.55, 2.27)	**1.86** (1.45, 2.38)	**1.37** (1.23, 1.53)	**1.61** (1.45, 1.77)	**1.68** (1.47, 1.91)	**1.62** (1.26, 2.08)	**1.77** (1.35, 2.32)	**2.08** (1.58, 2.75)
Level of discrimination for each race/ethnicity: Models 10–18									
Non-Hispanic White only × discrimination	**2.48** (1.72, 3.56)	**2.32** (1.77, 3.05)	**2.33** (1.67, 3.26)	**1.34** (1.11, 1.63)	**1.71** (1.44, 2.02)	**1.61** (1.30, 2.01)	1.27 (0.76, 2.15)	**1.87** (1.19, 2.93)	1.72 (0.86, 3.42)
Non-Hispanic Black only × discrimination	0.91 (0.44, 1.90)	1.22 (0.85, 1.74)	1.38 (0.91, 2.08)	**1.21** (1.01, 1.45)	**1.36** (1.15, 1.61)	**1.57** (1.26, 1.94)	**1.66** (1.15, 2.40)	**1.75** (1.21, 2.54)	**2.58** (1.86, 3.58)
Hispanic only × discrimination	**1.63** (1.07, 2.46)	**1.82** (1.33, 2.51)	**1.79** (1.14, 2.81)	**1.42** (1.17, 1.71)	**1.50** (1.27, 1.78)	**1.55** (1.23, 1.95)	**1.77** (1.26, 2.49)	1.63 (0.94, 2.82)	**1.77** (1.02, 3.09)
Non-Hispanic Asian only × discrimination	0.85 (0.03, 26.59)	0.63 (0.02, 21.48)	1.35 (0.07, 26.82)	1.20 (0.63, 2.27)	1.82 (1.00, 3.32)	1.37 (0.45, 4.17)	5.93 (1.89, 18.61)	3.01 (0.26, 34.17)	3.22 (0.27, 38.31)
Non-Hispanic other race or multiracial only × discrimination	**1.60** (1.03, 2.48)	**1.91** (1.32, 2.78)	**1.93** (1.27, 2.94)	**1.49** (1.17, 1.91)	**1.80** (1.47, 2.20)	**2.02** (1.55, 2.62)	**1.93** (1.14, 3.28)	**2.04** (1.34, 3.11)	**2.01** (1.17, 3.45)

*Notes:* Boldface indicates statistical significance (*p*<0.05).

All models are adjusted for time, the respondent’s age at baseline, the respondent’s sex, parental marital status, parental education level, parental employment status, and total combined family income. All outcomes are time varying; all covariates (except time) are time invariant. Main effects for race/ethnicity and discrimination were not included in Models 10–18. CIs are adjusted for multiple comparisons using the Bonferroni method, with m=15 (m=the number of effects in each group of models, e.g., the curiosity group of main effect models).

obs., observations.

## References

[1] Substance Abuse and Mental Health Services Administration (SAMHSA). Key substance use and mental health indicators in the United States: results from the 2021 National Survey on Drug Use and Health (HHS publication No. PEP22-07-01-005, NSDUH Series H-57). Bethesda, MD: Substance Abuse and Mental Health Services Administration (SAMHSA); 2022. https://www.samhsa.gov/data/report/2021-nsduh-annual-national-report. Published Accessed December 22, 2024.

[2] Substance Abuse and Mental Health Services Administration (SAMHSA). Key substance use and mental health indicators in the United States: results from the 2023 National Survey on Drug Use and Health (HHS publication No. PEP24-07-021, NSDUH Series H-59). Bethesda, MD: Substance Abuse and Mental Health Services Administration (SAMHSA); 2024. https://www.samhsa.gov/data/report/2023-nsduh-annual-national-report. Published Accessed XXX.

[3] Centers for Disease Control and Prevention [CDC], The Health Consequences of Smoking: 50 Years of Progress. A Report of the Surgeon General https://www.ncbi.nlm.nih.gov/books/NBK179276/. 24455788

[4] HHS. Smoking cessation. a report of the Surgeon General. Atlanta, GA: HHS, Centers for Disease Control and Prevention, National Center for Chronic Disease, Prevention and Health Promotion, Office on Smoking and Health; 2020. https://www.hhs.gov/sites/default/files/2020-cessation-sgr-full-report.pdf. Published Accessed XXX.

[5] Miech RA, Johnston LD, Patrick ME, O’Malley PM, Bachman JG, Schulenberg JE. Monitoring the Future national survey results on drug use, 1975–2022: secondary school students. Ann Arbor, MI: Institute for Social Research, University of Michigan, Monitoring the Future; 2023. https://monitoringthefuture.org/wp-content/uploads/2022/12/mtf2022.pdf. Published Accessed December 22, 2024.

[6] Miech RA, Johnston LD, Patrick ME, O’Malley PM, Bachman JG. Monitoring the Future National Survey Results on Drug Use, 1975–2023: Overview and Detailed Results for Secondary School Students. Ann Arbor, MI: Institute for Social Research, University of Michigan; 2024. https://monitoringthefuture.org/wp-content/uploads/2023/12/mtf2023.pdf. Published Accessed December 22, 2024.

[7] Jamal A, Park-Lee E, Birdsey J, Tobacco product use among middle and high school students - national youth tobacco survey, United States, 2024. MMWR Morb Mortal Wkly Rep. 2024;73(41):917–924. 10.15585/mmwr.mm7341a2. 39418216 PMC11486349

[8] King KM, Chassin L. A prospective study of the effects of age of initiation of alcohol and drug use on young adult substance dependence. J Stud Alcohol Drugs. 2007;68(2):256–265. 10.15288/jsad.2007.68.256. 17286344

[9] Lynskey MT, Heath AC, Bucholz KK, Escalation of drug use in early-onset cannabis users vs co-twin controls. JAMA. 2003;289 (4):427–433. 10.1001/jama.289.4.427. 12533121

[10] Myers HF. Ethnicity- and socio-economic status-related stresses in context: an integrative review and conceptual model. J Behav Med. 2009;32(1):9–19. 10.1007/s10865-008-9181-4. 18989769

[11] Okamoto J, Ritt-Olson A, Soto D, Baezconde-Garbanati L, Unger JB. Perceived discrimination and substance use among Latino adolescents. Am J Health Behav. 2009;33(6):718–727. 10.5993/ajhb.33.6.9. 19320620 PMC3769114

[12] Gibbons FX, Gerrard M, Cleveland MJ, Wills TA, Brody G. Perceived discrimination and substance use in African American parents and their children: a panel study. J Pers Soc Psychol. 2004;86(4):517–529. 10.1037/0022-3514.86.4.517. 15053703

[13] Brody GH, Kogan SM, Chen Y-F. Perceived discrimination and longitudinal increases in adolescent substance use: gender differences and mediational pathways. Am J Public Health. 2012;102(5):1006–1011. 10.2105/AJPH.2011.300588. 22420807 PMC3325367

[14] Gibbons FX, O’Hara RE, Stock ML, Gerrard M, Weng CY, Wills TA. The erosive effects of racism: reduced self-control mediates the relation between perceived racial discrimination and substance use in African American adolescents. J Pers Soc Psychol. 2012;102(5):1089–1104. 10.1037/a0027404. 22390225 PMC3341491

[15] Unger JB, Schwartz SJ, Huh J, Soto DW, Baezconde-Garbanati L. Acculturation and perceived discrimination: predictors of substance use trajectories from adolescence to emerging adulthood among Hispanics. Addict Behav. 2014;39(9):1293–1296. 10.1016/j.addbeh.2014.04.014. 24837753 PMC4060241

[16] Song J, Ip KI, Yan J, Lui PP, Kamata A, Kim SY. Pathways linking ethnic discrimination and drug-using peer affiliation to underage drinking status among Mexican-origin adolescents. Exp Clin Psychopharmacol. 2022;30(5):609–619. 10.1037/pha0000504. 34242039 PMC8861974

[17] Dai HD, Thiel G, Hafer D. Perceived racism and discrimination and youth substance use in the United States – intersections with sex and ethnicity. Prev Med. 2024;178:107811. 10.1016/j.ypmed.2023.107811. 38081420 PMC10928724

[18] Gibbons FX, Etcheverry PE, Stock ML, Exploring the link between racial discrimination and substance use: what mediates? What buffers? J Pers Soc Psychol. 2010;99(5):785–801. 10.1037/a0019880. 20677890 PMC3314492

[19] Basáñez T, Unger JB, Soto D, Crano W, Baezconde-Garbanati L. Perceived discrimination as a risk factor for depressive symptoms and substance use among Hispanic adolescents in los Angeles. Ethn Health. 2013;18(3):244–261. 10.1080/13557858.2012.713093. 22897755 PMC4511266

[20] Wedel AV, Goodhines PA, Zaso MJ, Park A. Prospective associations of discrimination, race, and sexual orientation with substance use in adolescents. Subst Use Misuse. 2022;57(2):263–272. 10.1080/10826084.2021.2002904. 34809528 PMC9132580

[21] Tao X, Liu T, Fisher CB, Giorgi S, Curtis B. COVID-related social determinants of substance use disorder among diverse U.S. racial ethnic groups. Soc Sci Med. 2023;317:115599. 10.1016/j.socscimed.2022.115599. 36525785 PMC9721390

[22] Leventhal AM, Cho J, Andrabi N, Barrington-Trimis J. Association of reported concern about increasing societal discrimination with adverse behavioral health outcomes in late adolescence. JAMA Pediatr. 2018;172(10):924–933. 10.1001/jamapediatrics.2018.2022. 30128537 PMC6233761

[23] Pierce JP, Choi WS, Gilpin EA, Farkas AJ, Merritt RK. Validation of susceptibility as a predictor of which adolescents take up smoking in the United States. Health Psychol. 1996;15(5):355–361. 10.1037//0278-6133.15.5.355. 8891714

[24] Strong DR, Hartman SJ, Nodora J, Predictive validity of the expanded susceptibility to smoke index. Nicotine Tob Res. 2015;17 (7):862–869. 10.1093/ntr/ntu254. 25481915 PMC4481694

[25] Ajzen I The theory of planned behavior. Organ Behav Hum Decis Process. 1991;50(2):179–211. 10.1016/0749-5978(91)90020-T.

[26] Marcoux BC, Shope JT. Application of the Theory of Planned Behavior to adolescent use and misuse of alcohol. Health Educ Res. 1997;12(3):323–331. 10.1093/her/12.3.323.

[27] Jackson KM, Roberts ME, Colby SM, Barnett NP, Abar CC, Merrill JE. Willingness to drink as a function of peer offers and peer norms in early adolescence. J Stud Alcohol Drugs. 2014;75(3):404–414. 10.15288/jsad.2014.75.404. 24766752 PMC4002854

[28] Choi WS, Gilpin EA, Farkas AJ, Pierce JP. Determining the probability of future smoking among adolescents. Addiction. 2001;96(2):313–323. 10.1046/j.1360-0443.2001.96231315.x. 11182877

[29] Gritz ER, Prokhorov AV, Hudmon KS, Predictors of susceptibility to smoking and ever smoking: a longitudinal study in a triethnic sample of adolescents. Nicotine Tob Res. 2003;5(4):493–506. 10.1080/1462220031000118568. 12959787

[30] Jackson C Cognitive susceptibility to smoking and initiation of smoking during childhood: a longitudinal study. Prev Med. 1998;27(1):129–134. 10.1006/pmed.1997.0255. 9465363

[31] Kam JA, Matsunaga M, Hecht ML, Ndiaye K. Extending the theory of planned behavior to predict alcohol, tobacco, and marijuana use among youth of Mexican heritage. Prev Sci. 2009;10(1):41–53. 10.1007/s11121-008-0110-0. 18985451

[32] Maher RA, Rickwood D. The theory of planned behavior, domain specific self-efficacy and adolescent smoking. J Child Adolesc Subst Abuse. 1998;6(3):57–76. 10.1300/J029v06n03_04.

[33] Nodora J, Hartman SJ, Strong DR, Curiosity predicts smoking experimentation independent of susceptibility in a U.S. national sample. Addict Behav. 2014;39(12):1695–1700. 10.1016/j.addbeh.2014.06.002. 25117844 PMC4183504

[34] Malmberg M, Overbeek G, Vermulst AA, Monshouwer K, Vollebergh WAM, Engels RCME. The theory of planned behavior: precursors of marijuana use in early adolescence? Drug Alcohol Depend. 2012;123(1–3):22–28. 10.1016/j.drugalcdep.2011.10.011. 22056217

[35] Pierce JP, Distefan JM, Kaplan RM, Gilpin EA. The role of curiosity in smoking initiation. Addict Behav. 2005;30(4):685–696. 10.1016/j.addbeh.2004.08.014. 15833574

[36] Wade NE, Palmer CE, Gonzalez MR, Risk factors associated with curiosity about alcohol use in the ABCD cohort. Alcohol. 2021;92:11–19. 10.1016/j.alcohol.2021.01.002. 33434614 PMC8026718

[37] Wang Y, Zhao Z, Zhang MR, Sleep as a protective factor: multiple forms of discrimination and substance use intention among racially and ethnically Minoritized United States youth. J Adolesc Health. 2025;76(4):718–726. 10.1016/j.jadohealth.2024.12.004. 39945682 PMC11930617

[38] Wang Y, Zhang Y, Zhao Z, Multiple discrimination and substance use intention in late childhood: findings from the Adolescent Brain Cognitive Development (ABCD) study. J Adolesc Health. 2024;74(6):1217–1224. 10.1016/j.jadohealth.2024.01.028. 38483374 PMC11102326

[39] Kulis S, Marsiglia FF, Nieri T. Perceived ethnic discrimination versus acculturation stress: influences on substance use among latino youth in the southwest. J Health Soc Behav. 2009;50(4):443–459. 10.1177/002214650905000405. 20099450 PMC2821707

[40] Stock ML, Gibbons FX, Walsh LA, Gerrard M. Racial identification, racial discrimination, and substance use vulnerability among African American young adults. Pers Soc Psychol Bull. 2011;37(10):1349–1361. 10.1177/0146167211410574. 21628598 PMC4909367

[41] Garavan H, Bartsch H, Conway K, Recruiting the ABCD sample: design considerations and procedures. Dev Cogn Neurosci. 2018;32:16–22. 10.1016/j.dcn.2018.04.004. 29703560 PMC6314286

[42] Phinney JS, Madden T, Santos LJ. Psychological variables as predictors of perceived ethnic discrimination among minority and immigrant adolescents. J Appl Soc Psychol. 1998;28(11):937–953. 10.1111/j.1559-1816.1998.tb01661.x.

[43] Cano MA, Schwartz SJ, Castillo LG, Depressive symptoms and externalizing behaviors among Hispanic immigrant adolescents: examining longitudinal effects of cultural stress. J Adolesc. 2015;42 (1):31–39. 10.1016/j.adolescence.2015.03.017. 25899132 PMC4464969

[44] Garcia-Huidobro D, Davey C, Svetaz MV, Allen M. Validity and reliability of alcohol and marijuana use susceptibility among Latino youth. J Adolesc Health. 2017;60(2):S32–S33. 10.1016/j.jadohealth.2016.10.081.

[45] Stover PJ, Harlan WR, Hammond JA, Hendershot T, Hamilton CM. PhenX: a toolkit for interdisciplinary genetics research. Curr Opin Lipidol. 2010;21(2):136–140. 10.1097/MOL.0b013e3283377395. 20154612 PMC8038872

[46] Hai AH, Lopez-Quintero C, Elton A, Curran L, Bo A. The independent and joint effect of socioeconomic status and Multiracial status on the prevalence and frequency of substance use and depression among U.S. adolescents. Addict Behav. 2024;151:107953. 10.1016/j.addbeh.2024.107953. 38232635 PMC11446446

[47] Goings TC, Salas-Wright CP, Howard MO, Vaughn MG. Substance use among bi/multiracial youth in the United States: profiles of psychosocial risk and protection. Am J Drug Alcohol Abuse. 2018;44 (2):206–214. 10.1080/00952990.2017.1359617. 29053377 PMC7590899

[48] Jackson KF, Lecroy CW. The influence of race and ethnicity on substance use and negative activity involvement among monoracial and multiracial adolescents of the Southwest. J Drug Educ. 2009;39(2):195–210. 10.2190/DE.39.2.f. 19999705

[49] Clark TT, Nguyen AB. Family factors and mediators of substance use among African American adolescents. J Drug Issues. 2012;42(4):358–372. 10.1177/0022042612461770. 30760939 PMC6369686

[50] Myers LL. Substance use among rural African American adolescents: identifying risk and protective factors. Child Adolesc Soc Work J. 2013;30(1):79–93. 10.1007/s10560-012-0280-2.

[51] Wallace JM, Muroff JR. Preventing substance abuse among African American children and youth: race differences in risk factor exposure and vulnerability. J Prim Prev. 2002;22(3):235–261. 10.1023/A:1013617721016.

[52] Iwamoto DK, Kaya A, Grivel M, Clinton L. Under-researched demographics: heavy episodic drinking and alcohol-related problems among Asian Americans. Alcohol Res. 2016;38(1):17–25. 27159808 10.35946/arcr.v38.1.03 PMC4872609

[53] Keum BT, Choi AY. COVID-19 racism, depressive symptoms, drinking to cope motives, and alcohol use severity among Asian American emerging adults. Emerg Adulthood. 2022;10(6):1591–1601. 10.1177/21676968221117421. 38603255 PMC9353315

[54] Liu MA, Fox T, Salyers M, Zapolski T, Cyders MA. A meta-analysis on racial discrimination and alcohol use among Asian Americans. Alcohol Clin Exp Res (Hoboken). 2024;48(12):2207–2221. 10.1111/acer.15475. 39523467

[55] Iwamoto DK, Kane JC, Negi NJ, Collado A, Tofighi D. Racial discrimination, distress, coping motives, and alcohol-related problems among U.S.-born Asian American young adults. Asian Am J Psychol. 2022;13 (2):177–184. 10.1037/aap0000238.

[56] Le TP, Iwamoto DK. A longitudinal investigation of racial discrimination, drinking to cope, and alcohol-related problems among underage Asian American college students. Psychol Addict Behav. 2019;33(6):520–528. 10.1037/adb0000501. 31414850

[57] Hahm HC, Lahiff M, Guterman NB. Asian American adolescents’ acculturation, binge drinking, and alcohol- and tobacco-using peers. J Community Psychol. 2004;32(3):295–308. 10.1002/jcop.20002.

[58] Jelsma E, Varner F. African American adolescent substance use: the roles of racial discrimination and peer pressure. Addict Behav. 2020;101:106154. 10.1016/j.addbeh.2019.106154. 31645003 PMC6916719

[59] Ahuja M, Haeny AM, Sartor CE, Bucholz KK. Perceived racial and social class discrimination and cannabis involvement among Black youth and young adults. Drug Alcohol Depend. 2022;232:109304. 10.1016/j.drugalcdep.2022.109304. 35124388 PMC10228548

[60] Hacker KJ, Chen-Sankey J, Leventhal AM, Choi K. Concern for police brutality, societal discrimination, and school shootings and subsequent cigarette and cannabis use in los Angeles County Hispanic and non-Hispanic White youth: a longitudinal study. J Racial Ethn Health Disparities. 2024;11(6):3336–3345. 10.1007/s40615-023-01787-z. 37725252

[61] Assari S Racial differences in biopsychosocial pathways to tobacco and marijuana use among youth. J Racial Ethn Health Disparities. 2024. 10.1007/s40615-024-02035-8. PMC12241214 38807026

[62] Neblett EW Jr, Rivas-Drake D, Umaña-Taylor AJ. The promise of racial and ethnic protective factors in promoting ethnic minority youth development. Child Dev Perspectives. 2012;6(3):295–303. 10.1111/j.1750-8606.2012.00239.x.

[63] Harris-Britt A, Valrie CR, Kurtz-Costes B, Rowley SJ. Perceived racial discrimination and self-esteem in African American youth: racial socialization as a protective factor. J Res Adolesc. 2007;17(4):669–682. 10.1111/j.1532-7795.2007.00540.x. 20700391 PMC2917995

[64] AJ Umaña-Taylor, BM Tynes, RB Toomey, DR Williams, Mitchell KJ. Latino adolescents’ perceived discrimination in online and offline settings: an examination of cultural risk and protective factors. Dev Psychol. 2015;51(1):87–100. 10.1037/a0038432. 25546597 PMC4752111

[65] Scott SM, Wallander JL, Cameron L. Protective mechanisms for depression among racial/ethnic minority youth: empirical findings, issues, and recommendations. Clin Child Fam Psychol Rev. 2015;18 (4):346–369. 10.1007/s10567-015-0188-4. 26374228

[66] Brodish AB, Cogburn CD, Fuller-Rowell TE, Peck S, Malanchuk O, Eccles JS. Perceived racial discrimination as a predictor of health behaviors: the moderating role of gender. Race Soc Probl. 2011;3 (3):160–169. 10.1007/s12552-011-9050-6. 22844386 PMC3403827

[67] Lei Y, Shah V, Biely C, Discrimination and subsequent mental health, substance use, and well-being in young adults. Pediatrics. 2021;148(6):e2021051378. 10.1542/peds.2021-051378. PMC9126825 34816276

