# Supplementary material for: Ethnic Discrimination’s Role on Increased Substance Susceptibility and Use Among U.S. Youth
Source: Am J Prev Med. Author manuscript; Available in PMC 2026 May 1. (PMC13134643; doi:10.1016/j.amepre.2025.107956)
Supplement: 1 [file NIHMS2168592-supplement-1.docx]

**Ethnic Discrimination’s Role on Increased Substance Susceptibility and Use Among US Youth**

Robert Rosales, PhD, LCSW, Philip Veliz, PhD, John Jardine, MA, Alexander S. Weigard, PhD, Sean Esteban McCabe, PhD

American Journal of Preventive Medicine

**Appendix**

**Study Sample (Continued)**

The recruitment produced a baseline cohort who reflected the demographic and geographic diversity of the US adolescent population, which resulted in an oversampling of Black children and rural inhabitants. The ABCD consortium goal is to achieve 87% retention. The ABCD protocol is a comprehensive set of in-person physical, cognitive, social, emotional, environmental, behavioral, and academic assessments, as well as multimodal neuroimaging and biospecimen collection for hormonal, genetic, epigenetic, environmental exposure, and substance use analysis. In-person assessments are done annually (6-7 hours for the adolescent and 3 hours for the parent) or biannually (imaging, bioassays) for 10 years. Participants also undergo a brief, mid-year phone interview. The ABCD uses an arsenal of measures from well- established assessments that include (but not limited to) the Child Behavior Checklist (CBCL), the NIH Toolbox Cognition Battery, and the substance use module that includes multiple validated measures.

**Results (Continued)**

Compared to White youth, Black youth reported significantly lower lifetime use of alcohol (aOR=.26, 95% CI=.11-.59) and tobacco (aOR=.44, 95% CI=.27-.71), lower curiosity towards alcohol (aOR=.76, 95% CI=.61-.94) and tobacco (aOR=.74, 95% CI=.60-.92), and higher willingness to try alcohol (aOR=1.83, 95% CI=1.08-3.10). Hispanic youth only reported significantly higher willingness to try alcohol than White youth (aOR=2.03, 95% CI=1.25-3.29). Asian youth only reported significantly lower lifetime tobacco use when compared with White youth (aOR=.11, 95% CI=.01-.87). There were no statistically significant differences between White and other race/multiracial youth in terms of substance use and susceptibility.

Higher levels of ethnic discrimination were significantly associated with increased odds of ever using alcohol (aOR=1.93, 95% CI=1.46-2.52), tobacco (aOR=1.88, 95% CI=1.55-2.27), and cannabis (aOR=1.86, 95% CI =1.45-2.38). Higher levels of ethnic discrimination were also associated with greater curiosity (alcohol: aOR=1.37, 95% CI=1.23-1.53; tobacco: aOR=1.61, 95% CI=1.45-1.77; cannabis: aOR=1.68, 95% CI=1.47, 1.91) and willingness to use (alcohol: aOR=1.62, 95% CI=1.26-2.08; tobacco: aOR=1.77, 95% CI=1.35-2.32; cannabis: aOR=2.08, 95% CI=1.58-2.75) each substance type in this study, after adjusting for relevant covariates.

Table 3 also presents the analysis assessing level of discrimination for each race/ethnic group and lifetime substance susceptibility and use. Higher levels of ethnic discrimination were consistently associated with greater odds of ever using, curiosity, and willingness to use substances among all race/ethnic groups in this study. White youth who reported greater discrimination reported greater lifetime use of alcohol (aOR=2.48, 95% CI=1.72-3.56), tobacco (aOR=2.32, 95% CI=1.77-3.05), and cannabis (aOR=2.33, 95% CI=1.67-3.26). They also reported greater curiosity to all substances in this study (alcohol: aOR=1.34, 95% CI=1.11-1.63; tobacco: aOR=1.71, 95% CI=1.44-2.02; cannabis: aOR=1.61, 95% CI=1.30-2.01). White youth also reported significantly greater odds of being willing to try tobacco (aOR=1.87, 95% CI=1.19, 2.93), but willingness to try alcohol or cannabis were not statistically significantly related to discrimination among this group.

Among Black youth, higher discrimination was significantly related with greater curiosity towards alcohol (aOR=1.21, 95% CI=1.01-1.45), tobacco (aOR=1.36, 95% CI=1.15-1.61), and cannabis (aOR=1.57, 95% CI=1.26-1.94). They also reported significantly greater odds of willingness to try alcohol (aOR=1.66, 95% CI=1.15-2.40), tobacco (aOR=1.75, 95% CI=1.21-2.54), and cannabis (aOR=2.58, 95% CI=1.86-3.58) when experiencing discrimination. However, discrimination was not significantly related with lifetime use of alcohol, tobacco, or cannabis among Black youth.

Latinx youth with who experienced greater discrimination had significantly higher odds of curiosity towards alcohol (aOR=1.63, 95% CI=1.07-2.46), tobacco (aOR=1.82, 95% CI=1.33-2.51), and cannabis (aOR=1.79, 95% CI=1.14-2.81). They also reported significantly greater odds of curiosity towards alcohol (aOR=1.42, 95% CI=1.17-1.71), tobacco (aOR=1.50, 95% CI=1.27-1.78), and cannabis (aOR=1.55, 95% CI=1.23-1.95) when experiencing discrimination. Greater discrimination among this group was also related with greater odds of willingness to try alcohol (aOR=1.77, 95% CI=1.26-2.49) and cannabis (aOR=1.77, 95% CI=1.02-3.09), but not tobacco.

Among Asian youth, great discrimination was only significantly associated with greater odds of willingness to try alcohol (aOR=5.93, 95% CI=1.89-18.61). However, this finding should be interpreted with cautious, considering the wide confidence intervals.

The analysis assessing level of discrimination for each race/ethnicity also found that discrimination for other/multiracial youth was related with significantly greater odds of lifetime susceptibility and use of all the substances in this study. Specifically, discrimination was related with greater odds of ever using alcohol (aOR=1.60, 95% CI=1.03, 2.48), tobacco (aOR=1.91, 95% CI=1.32, 2.78), and cannabis (aOR=1.93, 95% CI=1.27, 2.94). Discrimination was also related with greater odds of curiosity towards all the substances assessed in this study (alcohol: aOR=1.49, 95% CI=1.17-1.91; tobacco: aOR=1.80, 95% CI=1.47-2.20; cannabis: aOR=2.02, 95% CI=1.55-2.62). Finally, discrimination was associated with greater odds of being willing to try alcohol (aOR=1.93, 95% CI=1.14-3.28), tobacco (aOR=2.04, 95% CI=1.34-3.11), and cannabis (aOR=2.01, 95% CI=1.17-3.45) among this group.

**Appendix Table 1:** Study Sample Characteristics of Respondents in the ABCD Study by Wave: n (%)

| **Variable** | **Response Category** | **Baseline** | **Follow-up 1** | **Follow-up 2** | **Follow-up 3** | **Follow-up 4** |
| --- | --- | --- | --- | --- | --- | --- |
| Highest level of parental education | Less than high school | 433 (3.65) | 383 (3.42) | 362 (3.30) | 314 (3.04) | 132 (2.78) |
|  | High school | 1292 (10.90) | 1152 (10.28) | 1125 (10.26) | 998 (9.66) | 404 (8.51) |
|  | Some college | 1505 (12.70) | 1376 (12.28) | 1347 (12.29) | 1233 (11.94) | 573 (12.07) |
|  | Associate’s degree | 1569 (13.24) | 1470 (13.12) | 1422 (12.97) | 1318 (12.76) | 623 (13.12) |
|  | Bachelor’s degree or higher | 7055 (59.52) | 6827 (60.91) | 6704 (61.17) | 6463 (62.59) | 3015 (63.51) |
|  | Missing | 14 (0.12) | 12 (0.11) | 13 (0.12) | 10 (0.10) | 7 (0.15) |
| Parental marital status | Not married | 3789 (32.19) | 3471 (31.15) | 3376 (30.97) | 3054 (29.72) | 1340 (28.31) |
|  | Married | 7983 (67.81) | 7672 (68.85) | 7526 (69.03) | 7222 (70.28) | 3393 (71.69) |
|  | Missing | 96 (0.81) | 77 (0.69) | 71 (0.65) | 60 (0.58) | 21 (0.44) |
| Parental employment status | Neither parent or partner works full-time | 1790 (15.20) | 1603 (14.39) | 1562 (14.33) | 1402 (13.65) | 597 (12.64) |
|  | At least one of parent or partner works full-time | 9984 (84.80) | 9533 (85.61) | 9336 (85.67) | 8867 (86.35) | 4127 (87.36) |
|  | Missing | 94 (0.79) | 84 (0.75) | 75 (0.68) | 67 (0.65) | 30 (0.63) |
| Total combined family income | $24,999 or lower | 1634 (13.77) | 1453 (12.95) | 1400 (12.76) | 1252 (12.11) | 530 (11.15) |
|  | $25,000 to 49,999 | 1588 (13.38) | 1479 (13.18) | 1459 (13.30) | 1356 (13.12) | 641 (13.48) |
|  | $50,000 to 74,999 | 1498 (12.62) | 1420 (12.66) | 1388 (12.65) | 1319 (12.76) | 619 (13.02) |
|  | $75,000 to 99,999 | 1570 (13.23) | 1520 (13.55) | 1505 (13.72) | 1426 (13.80) | 681 (14.32) |
|  | $100,000 or greater | 4561 (38.44) | 4432 (39.50) | 4343 (39.58) | 4193 (40.57) | 1933 (40.66) |
|  | Don’t know | 504 (4.25) | 454 (4.05) | 428 (3.90) | 391 (3.78) | 173 (3.64) |
|  | Refuse to answer | 511 (4.31) | 461 (4.11) | 450 (4.10) | 399 (3.86) | 177 (3.72) |
|  | Missing | 2 (0.02) | 1 (0.01) | 0 (0.00) | 0 (0.00) | 0 (0.00) |

**Appendix Table 2:** Prevalence of Discrimination Score by Wave

|  | **Discrimination Score: n (%)** | | | |
| --- | --- | --- | --- | --- |
| **Event** | **x = 0** | **0 < x ≤ 0.5** | **x > 0.5** | **Missing** |
| Baseline |  |  |  | 11,868 (100.00) |
| Follow-up 1 | 6,991 (64.23) | 2,646 (24.31) | 1,247 (11.46) | 336 (2.99) |
| Follow-up 2 | 7,583 (70.86) | 2,120 (19.81) | 999 (9.33) | 271 (2.47) |
| Follow-up 3 |  |  |  | 10,336 (100.00) |
| Follow-up 4 | 2,961 (63.46) | 1,053 (22.57) | 652 (13.97) | 88 (1.85) |

**Notes.** The discrimination item was not assessed at baseline or follow-up 3 in the ABCD study. Therefore, there is complete missingness at these time points.

**Appendix Table 3:** Prevalence of Substance Susceptibility and Use by Wave: n (%)

|  |  | **Substance Use** | | | **Curiosity** | | | **Will Try Soon** | | |
| --- | --- | --- | --- | --- | --- | --- | --- | --- | --- | --- |
| **Substance** | **Event** | **No** | **Yes** | **Missing** | **No** | **Yes** | **Missing** | **No** | **Yes** | **Missing** |
| Alcohol | Baseline | 11,837 (99.82) | 21 (0.18) | 10 (0.08) | 7,592 (89.39) | 901 (10.61) | 3,375 (28.44) | 8,337 (98.26) | 148 (1.74) | 3,383 (28.51) |
|  | Follow-up 1 | 11,187 (99.74) | 29 (0.26) | 4 (0.04) | 6,877 (87.62) | 972 (12.38) | 3,371 (30.04) | 7,770 (98.98) | 80 (1.02) | 3,370 (30.04) |
|  | Follow-up 2 | 10,908 (99.51) | 54 (0.49) | 11 (0.10) | 6,252 (84.01) | 1,190 (15.99) | 3,531 (32.18) | 7,355 (98.86) | 85 (1.14) | 3,533 (32.20) |
|  | Follow-up 3 | 10,187 (98.71) | 133 (1.29) | 16 (0.15) | 5,560 (83.65) | 1,087 (16.35) | 3,689 (35.69) | 6,558 (98.91) | 72 (1.09) | 3,706 (35.86) |
|  | Follow-up 4 | 4,573 (96.38) | 172 (3.62) | 9 (0.19) | 2,198 (75.95) | 696 (24.05) | 1,860 (39.12) | 2,821 (97.92) | 60 (2.08) | 1,873 (39.40) |
| Tobacco | Baseline | 11,741 (99.01) | 117 (0.99) | 10 (0.08) | 9,479 (90.28) | 1,020 (9.72) | 1,369 (11.54) | 10,601 (99.30) | 75 (0.70) | 1,192 (10.04) |
|  | Follow-up 1 | 11,131 (99.24) | 85 (0.76) | 4 (0.04) | 9,228 (89.99) | 1,027 (10.01) | 965 (8.60) | 10,293 (99.60) | 41 (0.40) | 886 (7.90) |
|  | Follow-up 2 | 10,839 (98.88) | 123 (1.12) | 11 (0.10) | 6,258 (89.50) | 734 (10.50) | 3,981 (36.28) | 7,043 (99.82) | 13 (0.18) | 3,917 (35.70) |
|  | Follow-up 3 | 10,071 (97.59) | 249 (2.41) | 16 (0.15) | 7,955 (83.23) | 1,603 (16.77) | 778 (7.53) | 9,443 (98.96) | 99 (1.04) | 794 (7.68) |
|  | Follow-up 4 | 4,501 (94.86) | 244 (5.14) | 9 (0.19) | 3,435 (77.93) | 973 (22.07) | 346 (7.28) | 4,338 (98.70) | 57 (1.30) | 359 (7.55) |
| Cannabis | Baseline | 11,842 (99.90) | 12 (0.10) | 14 (0.12) | 6,139 (97.58) | 152 (2.42) | 5,577 (46.99) | 6,317 (99.64) | 23 (0.36) | 5,528 (46.58) |
|  | Follow-up 1 | 11,165 (99.55) | 51 (0.45) | 4 (0.04) | 8,169 (96.36) | 309 (3.64) | 2,742 (24.44) | 8,506 (99.81) | 16 (0.19) | 2,698 (24.05) |
|  | Follow-up 2 | 10,896 (99.40) | 66 (0.60) | 11 (0.10) | 6,709 (94.31) | 405 (5.69) | 3,859 (35.17) | 7,100 (99.54) | 33 (0.46) | 3,840 (34.99) |
|  | Follow-up 3 | 10,208 (98.91) | 112 (1.09) | 16 (0.15) | 6,785 (91.18) | 656 (8.82) | 2,895 (28.01) | 7,373 (99.19) | 60 (0.81) | 2,903 (28.09) |
|  | Follow-up 4 | 4,561 (96.12) | 184 (3.88) | 9 (0.19) | 3,391 (82.29) | 730 (17.71) | 633 (13.32) | 4,014 (97.81) | 90 (2.19) | 650 (13.67) |

**Notes.** For a given event, only respondents who knew what the substance was, but hadn’t tried it yet (including just a sip of alcohol or a puff of tobacco / marijuana), were asked about their curiosity and willingness to try that substance.

**Appendix Table 4:** Adjusted Odds Ratios for Substance Susceptibility and Use as Functions of Race / Ethnicity x Discrimination (Time-Invariant): aOR (95% CI)

|  | **Substance Use** | | | **Susceptibility to Use** | | | | | |
| --- | --- | --- | --- | --- | --- | --- | --- | --- | --- |
|  |  | | | **Curiosity** | | | **Will Try Soon** | | |
|  | **Alcohol** | **Tobacco** | **Cannabis** | **Alcohol** | **Tobacco** | **Cannabis** | **Alcohol** | **Tobacco** | **Cannabis** |
|  | **n = 11,257** | **n = 11,257** | **n = 11,257** | **n = 8,657** | **n = 11,122** | **n = 10,905** | **n = 8,660** | **n = 11,121** | **n = 10,905** |
| **Full Models – Main Effects and Interaction Effects: Models 1-9** | **obs. =**  **47,834** | **obs. =**  **47,834** | **obs. =**  **47,831** | **obs. =**  **32,474** | **obs. =**  **40,713** | **obs. =**  **32,714** | **obs. =**  **32,421** | **obs. =**  **41,005** | **obs. =**  **32,794** |
| Non-Hispanic White only | Ref. | Ref. | Ref. | Ref. | Ref. | Ref. | Ref. | Ref. | Ref. |
| Non-Hispanic Black only | 0.28 (0.10, 0.81) | 0.51 (0.27, 0.95) | 1.00 (0.52, 1.94) | 0.72 (0.55, 0.94) | 0.75 (0.58, 0.98) | 0.75 (0.51, 1.09) | 2.03 (1.06, 3.90) | 1.34 (0.60, 3.02) | 1.22 (0.49, 3.04) |
| Hispanic | 0.93 (0.49, 1.75) | 0.82 (0.51, 1.33) | 1.14 (0.63, 2.08) | 1.17 (0.95, 1.43) | 1.04 (0.85, 1.27) | 0.81 (0.60, 1.10) | 2.25 (1.27, 3.98) | 1.29 (0.61, 2.73) | 1.07 (0.46, 2.53) |
| Non-Hispanic Asian only | NA | 0.02 (0.00, 23.30) | 0.05 (0.00, 114.83) | 0.77 (0.44, 1.35) | 0.81 (0.47, 1.42) | 0.48 (0.18, 1.29) | 0.82 (0.14, 4.70) | NA | NA |
| Non-Hispanic other race or multiracial | 0.81 (0.41, 1.60) | 1.21 (0.72, 2.05) | 1.01 (0.49, 2.09) | 0.91 (0.69, 1.19) | 0.99 (0.78, 1.27) | 1.15 (0.83, 1.60) | 1.20 (0.58, 2.51) | 1.43 (0.64, 3.23) | 1.66 (0.65, 4.25) |
| Discrimination score | 2.24 (1.46, 3.44) | 2.15 (1.56, 2.96) | 2.36 (1.62, 3.46) | 1.32 (1.06, 1.63) | 1.67 (1.38, 2.01) | 1.52 (1.19, 1.96) | 1.70 (1.05, 2.76) | 2.05 (1.25, 3.34) | 1.83 (0.84, 4.02) |
| Non-Hispanic Black only x Discrimination | 0.82 (0.37, 1.81) | 0.78 (0.47, 1.31) | 0.60 (0.32, 1.12) | 1.12 (0.82, 1.51) | 0.95 (0.72, 1.26) | 1.16 (0.80, 1.70) | 0.85 (0.42, 1.69) | 0.80 (0.40, 1.62) | 1.35 (0.56, 3.25) |
| Hispanic x Discrimination | 0.67 (0.35, 1.30) | 0.88 (0.53, 1.44) | 0.71 (0.36, 1.40) | 0.93 (0.67, 1.27) | 0.85 (0.64, 1.12) | 1.11 (0.76, 1.61) | 0.80 (0.41, 1.56) | 0.75 (0.32, 1.74) | 0.99 (0.35, 2.78) |
| Non-Hispanic Asian only x Discrimination | NA | 7.03 (0.09, 571.16) | 5.03 (0.03, 945.15) | 1.19 (0.52, 2.70) | 1.32 (0.59, 2.91) | 1.72 (0.44, 6.74) | 4.95 (0.73, 33.57) | NA | NA |
| Non-Hispanic other race or multiracial x Discrimination | 0.74 (0.38, 1.42) | 0.72 (0.40, 1.31) | 0.82 (0.42, 1.62) | 1.19 (0.82, 1.75) | 1.06 (0.77, 1.45) | 1.14 (0.76, 1.72) | 1.28 (0.58, 2.86) | 0.88 (0.43, 1.81) | 0.84 (0.29, 2.48) |

**Notes.** All models are adjusted for time, the respondent’s age at baseline, the respondent’s sex, parental marital status, parental education level, parental employment status, and total combined family income. All outcomes are time-varying; all covariates (except time) are time-invariant. Confidence intervals are adjusted for multiple comparisons using the Bonferroni method, with m = 27 (the number of effects in each group of models, e.g., the ‘curiosity to use’ group of models).

**Appendix Table 5:** Adjusted Odds Ratios for Substance Susceptibility and Use as Functions of Race / Ethnicity x Discrimination (Time-Varying): aOR (95% CI)

|  | **Substance Use** | | | **Susceptibility to Use** | | | | | |
| --- | --- | --- | --- | --- | --- | --- | --- | --- | --- |
|  |  | | | **Curiosity** | | | **Will Try Soon** | | |
|  | **Alcohol** | **Tobacco** | **Cannabis** | **Alcohol** | **Tobacco** | **Cannabis** | **Alcohol** | **Tobacco** | **Cannabis** |
|  | **n = 11,256** | **n = 11,256** | **n = 11,256** | **n = 8,188** | **n = 10,574** | **n = 9,901** | **n = 8,196** | **n = 10,605** | **n = 9,905** |
| **Full Models – Main Effects and Interaction Effects: Models 1-9** | **obs. =**  **25,888** | **obs. =**  **25,888** | **obs. =**  **25,888** | **obs. =**  **17,512** | **obs. =**  **20,931** | **obs. =**  **19,075** | **obs. =**  **17,491** | **obs. =**  **21,041** | **obs. =**  **19,113** |
| Non-Hispanic White only | Ref. | Ref. | Ref. | Ref. | Ref. | Ref. | Ref. | Ref. | Ref. |
| Non-Hispanic Black only | 0.19 (0.04, 0.82) | 0.77 (0.40, 1.48) | 0.88 (0.43, 1.81) | 0.80 (0.59, 1.07) | 0.77 (0.57, 1.06) | 0.83 (0.55, 1.23) | 1.77 (0.79, 3.96) | 1.57 (0.49, 5.04) | 1.60 (0.60, 4.23) |
| Hispanic | 0.66 (0.31, 1.39) | 0.88 (0.51, 1.50) | 1.05 (0.56, 1.99) | 1.16 (0.92, 1.45) | 1.00 (0.79, 1.27) | 0.76 (0.55, 1.05) | 1.62 (0.74, 3.52) | 1.39 (0.44, 4.36) | 0.88 (0.34, 2.29) |
| Non-Hispanic Asian only | 0.03 (0.00, 1.06) | 0.03 (0.00, 2.67) | 0.24 (0.01, 7.06) | 0.95 (0.53, 1.67) | 0.85 (0.46, 1.56) | 0.49 (0.18, 1.32) | 1.66 (0.31, 9.05) | NA | NA |
| Non-Hispanic other race or multiracial | 0.80 (0.37, 1.72) | 1.03 (0.56, 1.88) | 1.12 (0.54, 2.33) | 0.95 (0.71, 1.26) | 1.02 (0.77, 1.36) | 1.15 (0.80, 1.66) | 1.28 (0.51, 3.20) | 1.39 (0.39, 4.91) | 2.03 (0.77, 5.37) |
| Discrimination score^a^ | 2.54 (1.53, 4.22) | 2.31 (1.48, 3.63) | 2.77 (1.69, 4.53) | 1.55 (1.16, 2.08) | 2.04 (1.58, 2.63) | 1.66 (1.19, 2.32) | 1.63 (0.62, 4.29) | 2.66 (1.37, 5.18) | 2.21 (0.92, 5.26) |
| Non-Hispanic Black only x Discrimination | 0.99 (0.33, 2.97) | 0.60 (0.28, 1.26) | 0.63 (0.31, 1.27) | 1.05 (0.70, 1.57) | 0.88 (0.58, 1.32) | 1.06 (0.62, 1.82) | 0.99 (0.32, 3.08) | 0.72 (0.26, 2.00) | 0.94 (0.30, 2.94) |
| Hispanic x Discrimination | 0.97 (0.42, 2.23) | 0.91 (0.47, 1.75) | 0.77 (0.36, 1.63) | 0.96 (0.64, 1.46) | 0.90 (0.61, 1.32) | 1.35 (0.84, 2.16) | 1.06 (0.33, 3.43) | 0.88 (0.27, 2.88) | 1.30 (0.41, 4.15) |
| Non-Hispanic Asian only x Discrimination | 4.51 (1.68, 12.09) | 3.49 (0.44, 27.81) | 1.57 (0.05, 53.36) | 0.32 (0.05, 2.24) | 1.26 (0.44, 3.65) | 1.45 (0.32, 6.63) | 1.82 (0.08, 41.72) | NA | NA |
| Non-Hispanic other race or multiracial x Discrimination | 0.69 (0.29, 1.64) | 0.80 (0.38, 1.67) | 0.66 (0.27, 1.62) | 1.20 (0.73, 1.99) | 1.08 (0.68, 1.71) | 1.34 (0.77, 2.35) | 1.24 (0.34, 4.53) | 0.94 (0.30, 3.00) | 0.59 (0.15, 2.25) |

**Notes.** All models are adjusted for time, the respondent’s age at baseline, the respondent’s sex, parental marital status, parental education level, parental employment status, and total combined family income. All outcomes are time-varying; all covariates (except time and the discrimination item) are time-invariant. Confidence intervals are adjusted for multiple comparisons using the Bonferroni method, with m = 27 (the number of effects in each group of models, e.g., the ‘curiosity to use’ group of models).

^a^For all 9 models in this table, the discrimination item was kept as time-varying. Therefore, there are no observations from baseline or follow-up 3 included in these models due to complete missingness for the discrimination item.

**Appendix Table 6:** Adjusted Odds Ratios for Substance Susceptibility and Use as Functions of Race / Ethnicity and Discrimination (Time-Varying): aOR (95% CI)

|  | **Substance Use** | | | **Susceptibility to Use** | | | | | |
| --- | --- | --- | --- | --- | --- | --- | --- | --- | --- |
|  |  | | | **Curiosity** | | | **Will Try Soon** | | |
|  | **Alcohol** | **Tobacco** | **Cannabis** | **Alcohol** | **Tobacco** | **Cannabis** | **Alcohol** | **Tobacco** | **Cannabis** |
|  | **n = 11,256** | **n = 11,256** | **n = 11,256** | **n = 8,188** | **n = 10,574** | **n = 9,901** | **n = 8,196** | **n = 10,605** | **n = 9,905** |
| **Main Effects:**  **Models 1-9** | **obs. =**  **25,888** | **obs. =**  **25,888** | **obs. =**  **25,888** | **obs. =**  **17,512** | **obs. =**  **20,931** | **obs. =**  **19,075** | **obs. =**  **17,491** | **obs. =**  **21,041** | **obs. =**  **19,113** |
| Non-Hispanic White only | Ref. | Ref. | Ref. | Ref. | Ref. | Ref. | Ref. | Ref. | Ref. |
| Non-Hispanic Black only | 0.20 (0.07, 0.58) | 0.60 (0.34, 1.06) | 0.71 (0.38, 1.35) | 0.81 (0.62, 1.04) | 0.73 (0.56, 0.95) | 0.80 (0.58, 1.11) | 1.72 (0.86, 3.46) | 1.31 (0.49, 3.54) | 1.56 (0.71, 3.40) |
| Hispanic | 0.66 (0.36, 1.23) | 0.86 (0.55, 1.37) | 0.98 (0.57, 1.68) | 1.14 (0.93, 1.40) | 0.97 (0.79, 1.20) | 0.82 (0.62, 1.09) | 1.63 (0.84, 3.19) | 1.33 (0.49, 3.62) | 1.02 (0.47, 2.19) |
| Non-Hispanic Asian only | 0.11 (0.01, 2.13) | 0.10 (0.01, 1.91) | 0.34 (0.04, 2.90) | 0.79 (0.48, 1.30) | 0.91 (0.55, 1.52) | 0.54 (0.24, 1.23) | 1.95 (0.39, 9.67) | NA | NA |
| Non-Hispanic other race or multiracial | 0.65 (0.33, 1.29) | 0.96 (0.58, 1.61) | 0.96 (0.53, 1.75) | 0.99 (0.78, 1.27) | 1.06 (0.84, 1.34) | 1.25 (0.92, 1.69) | 1.39 (0.63, 3.07) | 1.41 (0.49, 3.99) | 1.57 (0.66, 3.72) |
| Discrimination score^a^ | 2.35 (1.70, 3.26) | 1.94 (1.51, 2.49) | 2.15 (1.63, 2.84) | 1.58 (1.37, 1.83) | 1.96 (1.70, 2.26) | 1.92 (1.61, 2.30) | 1.71 (1.21, 2.41) | 2.29 (1.52, 3.47) | 2.10 (1.40, 3.15) |
| **Level of Discrimination for**  **each Race/Ethnicity: Models 10-18** | | | | | | | | | |
| Non-Hispanic White only x Discrimination | 2.93 (1.86, 4.62) | 2.43 (1.63, 3.62) | 2.78 (1.79, 4.31) | 1.56 (1.19, 2.04) | 2.08 (1.64, 2.63) | 1.75 (1.28, 2.37) | 1.30 (0.48, 3.51) | 2.36 (1.24, 4.49) | 2.02 (0.90, 4.56) |
| Non-Hispanic Black only x Discrimination | 1.08 (0.40, 2.94) | 1.24 (0.74, 2.10) | 1.63 (1.03, 2.56) | 1.44 (1.13, 1.82) | 1.57 (1.21, 2.04) | 1.66 (1.19, 2.32) | 1.81 (1.12, 2.92) | 2.16 (1.18, 3.95) | 2.44 (1.38, 4.33) |
| Hispanic x Discrimination | 2.27 (1.30, 3.96) | 2.04 (1.35, 3.10) | 2.22 (1.37, 3.60) | 1.67 (1.28, 2.17) | 1.89 (1.47, 2.44) | 1.97 (1.47, 2.66) | 1.88 (1.09, 3.25) | 2.48 (1.05, 5.83) | 2.37 (1.18, 4.75) |
| Non-Hispanic Asian only x Discrimination | 1.03 (0.04, 27.40) | 0.81 (0.03, 21.03) | 1.42 (0.07, 28.12) | 0.47 (0.09, 2.50) | 2.25 (0.95, 5.35) | 1.28 (0.36, 4.58) | 3.74 (0.27, 52.14) | NA | NA |
| Non-Hispanic other race or multiracial x Discrimination | 1.74 (0.95, 3.16) | 1.98 (1.21, 3.24) | 1.98 (1.10, 3.56) | 1.81 (1.27, 2.57) | 2.28 (1.64, 3.18) | 2.57 (1.75, 3.78) | 1.93 (0.88, 4.23) | 2.68 (1.26, 5.73) | 1.89 (0.86, 4.13) |

**Notes.** All models are adjusted for time, the respondent’s age at baseline, the respondent’s sex, parental marital status, parental education level, parental employment status, and total combined family income. All outcomes are time-varying; all covariates (except time and the discrimination item) are time-invariant. Main effects for race / ethnicity and discrimination were not included in models 10-18. Confidence intervals are adjusted for multiple comparisons using the Bonferroni method, with m = 15 (m = the number of effects in each group of models, e.g., the ‘curiosity’ group of main effect models).

^a^For all 18 models in this table, the discrimination item was kept as time-varying. Therefore, there are no observations from baseline or follow-up 3 included in these models due to complete missingness for the discrimination item.
